# Molecular Diversity of Alkenal Double Bond Reductases in the Liverwort *Marchantia paleacea*

**DOI:** 10.3390/molecules23071630

**Published:** 2018-07-04

**Authors:** Yi-Feng Wu, Hong-Bo Zheng, Xin-Yan Liu, Ai-Xia Cheng, Hong-Xiang Lou

**Affiliations:** Key Laboratory of Chemical Biology of Natural Products, Ministry of Education, School of Pharmaceutical Sciences, Shandong University, Jinan 250012, China; wendywu.12@163.com (Y.-F.W.); zhenghongbo1991@126.com (H.-B.Z.); qqbailiu@163.com (X.-Y.L.)

**Keywords:** alkenal double bond reductase, *Marchantia paleacea*, hydroxycinnamyl aldehydes, microbial type, expression pattern

## Abstract

Alkenal double bond reductases (DBRs), capable of catalyzing the NADPH-dependent reduction of the α,β-unsaturated double bond, play key roles in the detoxication of alkenal carbonyls. Here, the isolation and characterization of two DBRs encoded by the liverwort species *Marchantia paleacea* are described. The two DBRs share a relatively low similarity, and phylogenetic analysis indicated that MpMDBRL is more closely related to microbial DBRs than to other plant DBRs, while MpDBR shares common ancestry with typical plant DBRs. Both DBR proteins exhibited hydrogenation ability towards hydroxycinnamyl aldehydes; however, their temperature optimums were strikingly different. MpMDBRL demonstrated slightly weaker catalytic efficiency compared to MpDBR, and the structural models of their active binding sites to the substrate may provide a parsimonious explanation. Furthermore, both DBRs significantly responded to phytohormone treatment. In conclusion, *M. paleacea* produces two distinct types of functional DBRs, both of which participate in the protection against environmental stress in liverwort. The presence of a microbial type of DBR in a plant is herein reported for the first time.

## 1. Introduction

Exposure of plant cells to abiotic and/or biotic stresses, such as pathogen attack, insect predation, and ultraviolet (UV) injury, often results in the production of toxic reactive compounds [1], including α,β-unsaturated carbonyls, which are involved in the pathophysiological effects associated with oxidative stress in cells and tissues [2]. The toxicity of these reactive aldehydes is due to the ability of their α,β-unsaturated bonds to form Michael adducts with thiol and amino groups in biomolecules [3]. As saturated forms lack this reactive moiety, the hydrogenation of the α,β-double bond by alkenal double bond reductases thus results in detoxication [4,5].

Genes encoding several reductases of this type from plants have been isolated. The product of the *Pinus taeda* gene *PtPPDBR* catalyzes the NADPH-dependent reduction of the α,β-unsaturated double bond of phenylpropenal aldehydes [6]. Its *Arabidopsis thaliana* homolog AtDBR1 (At5g16970) converts *p*-coumaryl aldehyde and coniferyl aldehyde into their corresponding dihydrophenylpropanols [7]. PaDBR1 and PaDBR2, isolated from the liverwort *Plagiochasma appendiculatum*, were characterized to exhibit hydrogenation ability towards hydroxycinnamyl aldehydes in our previous study [8]. The above enzymes belong to the zinc-independent, medium chain dehydrogenase/reductase (MDR) superfamily, and they all share a conserved GXXS motif, known to stabilize both the adenine and nicotinamide moieties of NADPH, along with a glycine-rich motif (either AXXGXXG or GXXGXXG) known to participate in the enzyme’s binding with the NAD(P)^+^ or NAD(P)H pyrophosphate [9].

Bryophytes (liverworts, mosses, and hornworts) grow on trees, in the soil, in lakes, in rivers, and even on Antarctic islands [10]. As the most primitive terrestrial plants [11,12], they are the pioneers to evolve ways to survive outside of the marine/aqueous environment. The transition to land entailed adaptation to a host of environmental challenges, requiring new survival mechanisms. The *Marchantia* genome shows evidence of substantial gene transfer from fungi and bacteria [13] via a mechanism where genetic material is moved across species other than by descent [14]. For example, *Marchantia polymorpha* microbial terpene synthase-like (MTPSL) genes involved in terpene biosynthesis appear to be the product of horizontal gene transfer from fungi [15,16,17]. To date, no microbial alkenal double bond reductase-like (MDBRL) genes have been identified in liverworts or other plants. Here, the isolation and functional characterization of two DBRs produced by the liverwort species *M. paleacea* are described. The striking difference between the two DBRs is that one is a typical plant DBR, whereas the other is microbial DBR-like. Their enzymatic characteristics, catalytic activities, and expression patterns were analyzed, and the results may shed light on the molecular diversity and evolution of double bond reductases in liverworts.

## 2. Results and Discussion

### 2.1. Isolation and Sequence Analysis of MpDBR and MpMDBRL

A search of the thallus transcriptome sequence datasets for *M. paleacea* (SRP078650) identified two candidate *DBR* homologs, namely MpDBR and MpMDBRL. The MpDBR sequence contained a 1026-bp ORF, putatively encoding a 341 amino acid polypeptide with a molecular mass of 37.98 kDa. The full-length MpMDBRL, as recovered by 3′-rapid amplification of cDNA ends (RACE) and 5′-RACE PCR, included a 1056-bp ORF predicted to encode a 351 residue polypeptide with a molecular mass of 38.75 kDa. Their full-length cDNA sequences have been deposited in GenBank as accessions MH427075 and MH427076. The two deduced polypeptides shared 42.82% of their identity with one another. In comparison to the high identity of 99.4% observed between PaDBR1 and PaDBR2 (both isolated from *P. appendiculatum*) [8], MpDBR and MpMDBRL share relatively low sequence similarity.

### 2.2. Sequence Alignment and Phylogenetic Analysis

**Figure 1:**
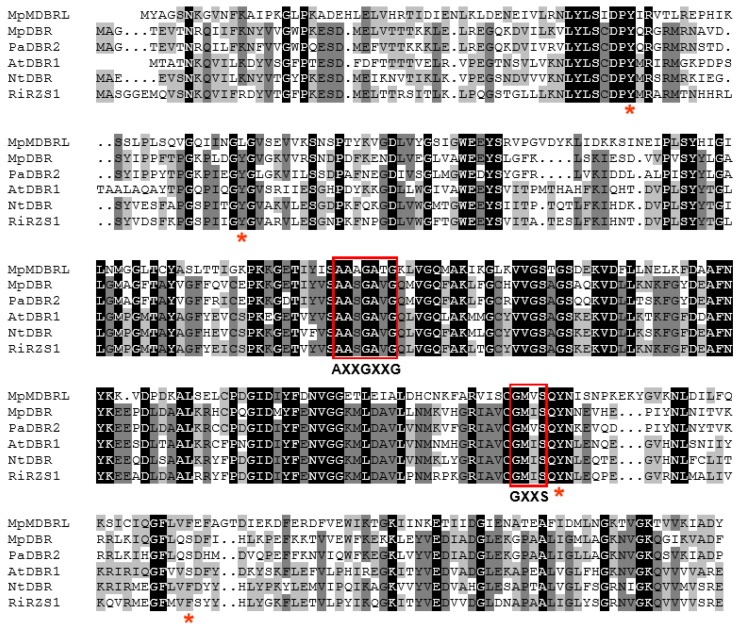
The peptide alignment of MpDBR and MpMDBRL with other double bond reductase sequences. PaDBR2 from *Plagiochasma appendiculatum*, AtDBR1 from *Arabidopsis thaliana*, NtDBR from *Nicotiana tabacum* and RiRZS1 from *Rubus idaeus*. Identical residues are shown in black and similar ones in gray. The conserved co-enzyme binding motifs AXXGXXG and GXXS are shown boxed, and active site residues indicated with an asterisk.

Both MpDBR and MpMDBRL harbored a conserved glycine-rich motif AASGAVG, as well as a GXXS motif. The MpDBR sequence shared 57.18%, 60.17%, and 58.33% identity with AtDBR1 (*A. thaliana* DBR1) [7], NtDBR (*Nicotiana tabacum* DBR) [18], and RiRZS1 (*Rubus idaeus* RZS1) [19], respectively, whereas the identity for MpMDBRL was 40.96%, 40.79%, and 43.14% (see Figure 1).

**Figure 2:**
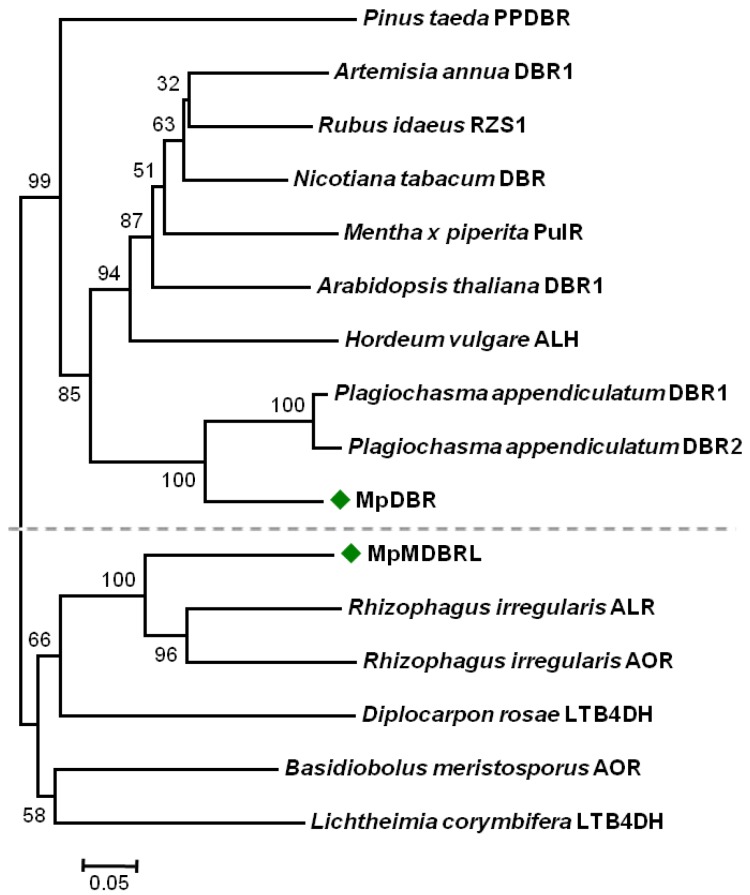
The phylogenetic analysis of MpDBR and MpMDBRL and other double bond reductase proteins from plants and microbes. The scale indicates evolutionary distance. The numbers shown are bootstrap values, based on 1000 replicates.

In order to elucidate the phylogenetic relationships of the DBR genes, a phylogenetic tree was constructed using characterized or putative DBRs from different organisms. The phylogenetic analysis showed that MpDBR and MpMDBRL are only distantly related. MpDBR was included in a clade containing typical higher plant DBRs, as well as two *P. appendiculatum* homologs (see Figure 2). In contrast, the protein sequence of MpMDBRL, categorized into another clade (see Figure 2), demonstrated greater similarity to microbial DBRs than MpDBR and other plant forms. As is the case for terpene synthases (TPS) in *Selaginella moellendorffii*, TPSs can be divided into two groups designated as *S. moellendorffii* TPS proteins (SmTPSs) and *S. moellendorffii* microbial TPS-like proteins (SmMTPSLs) [20]. Two types of DBRs may exist in *M. Paleacea* based on the phylogenetic analysis.

### 2.3. Functional Analysis

**Figure 3:**
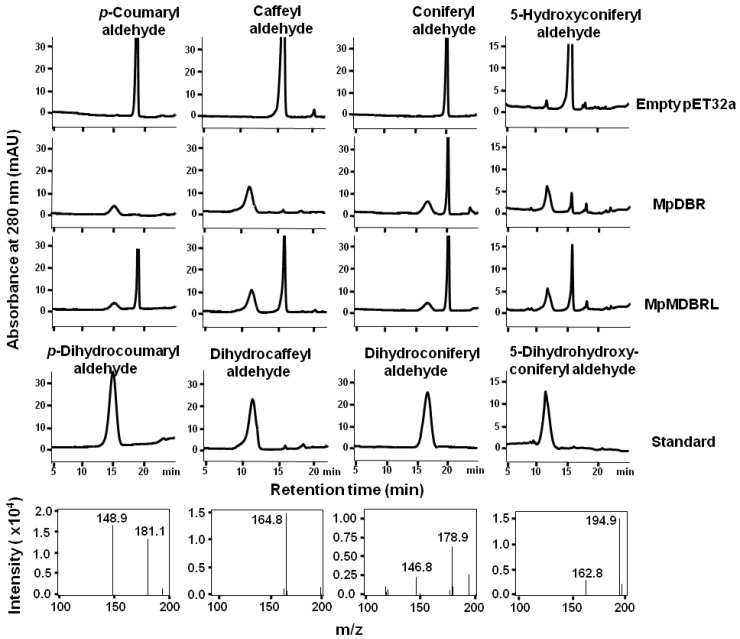
The HPLC profiles and MS spectra of reaction products generated by recombinant DBRs. The activities of recombinant DBRs when provided with either *p*-coumaryl, caffeyl, coniferyl, or 5-hydroxyconiferyl aldehyde as a substrate. The MS spectra of the MpDBR reaction products are shown in the lower panel. Negative ionization mode was used.

To further investigate the functional activity of DBRs in vitro, recombinant versions of DBR were expressed in the form of His-tagged fusions in *E. coli*. An analysis of the extracted proteins showed that each of the products was ~58 kDa in size (includes the 20.4-kDa His-tag), corresponding to the predicted masses (see Figure S1). As putative DBRs, hydroxycinnamyl aldehydes were tested using enzyme assays. MpDBR and MpMDBRL were able to accept *p*-coumaryl-, caffeyl-, coniferyl-, or 5-hydroxyconiferyl aldehyde as their substrate, and the major reaction products exhibited a similar retention time and molecular parent ion peak [M-H]^−^ as dihydro-*p*-coumaryl-, dihydrocaffeyl-, dihydroconiferyl-, and dihydro-5-hydroxyconiferyl aldehyde, respectively (see Figure 3). However, there was no evidence of reactivity when the two DBRs were provided with sinapyl aldehyde (data not shown).

**Figure 4:**
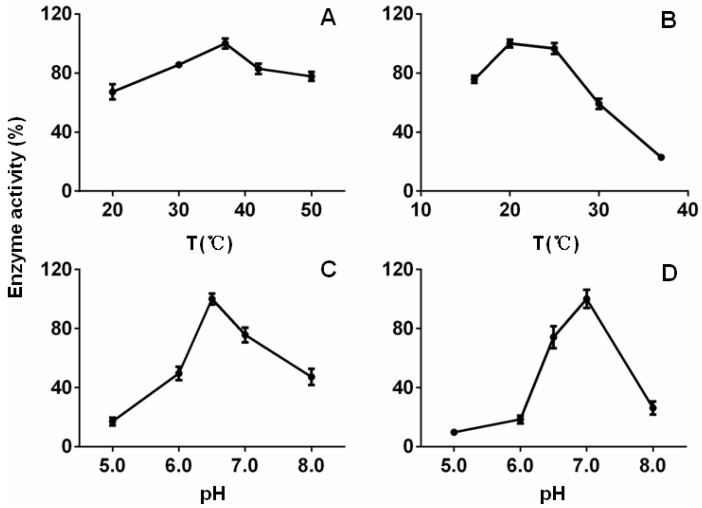
The optimal temperature and pH for the activity of recombinant DBRs. The effect of (**A**,**B**) temperature, (**C**,**D**) pH on (**A**,**C**) recombinant MpDBR, (**B**,**D**) recombinant MpMDBRL. All reactions contained caffeyl aldehyde. Data are shown in the form mean ± SD (*n* = 3).

An assessment of reductase activity revealed that the reaction pH of MpDBR generating the strongest activity was 6.5, and that of MpMDBRL was 7.0. Unexpectedly, the optimal temperature values for the two proteins seemed to be strikingly different. The temperature optimum for MpDBR was 37 °C, which is similar to most of the plant MDR family proteins, for instance, it was 37 °C for both DBRs from *P. appendiculatum* [8], 30 °C for AaDBR1 from *Artemisia annua* [21], and 30–40 °C for cinnamyl alcohol dehydrogenases (CADs) from *A. thaliana* [22]; whereas it was 20 °C for MpMDBRL, which is relatively lower. However, it was the same case for CjPAD, a phenolic acid decarboxylase (PAD) also from liverwort and related to microbial PAD, with temperature optima of 25 °C [23]. In addition, MpMDBRL was more temperature-sensitive (see Figure 4). These findings demonstrated that MpDBR and MpMDBRL appear to be two distinct types of DBRs.

**Figure 5:**
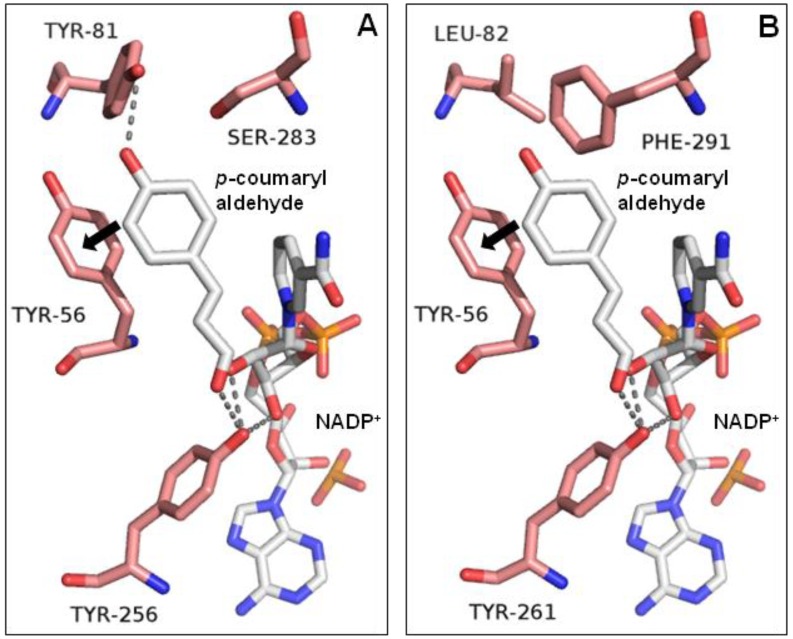
The active site of (**A**) MpDBR, (**B**) MpMDBRL in a complex with NADP^+^/*p*-coumaryl aldehyde. Arrows indicate the potential stacking interaction between phenol rings. Hydrogen bonds shown as dashed lines.

Under optimal conditions, MpDBR exhibited comparable catalytic efficiency towards four hydroxycinnamyl aldehydes, and MpMDBRL also behaved similarly towards each substrate. However, in vitro analyses demonstrated that MpMDBRL displayed slightly weaker catalytic behavior than MpDBR for each substrate (see Table 1). Structural models based on *A. thaliana* DBR were thus constructed in an attempt to explore this. The residues (and therefore the structure) surrounding the active center of MpDBR, Tyr56, Tyr81, Tyr256, and Ser283 matched the MpMDBRL residues, respectively, Tyr56, Leu82, Tyr261, and Phe291. According to Youn et al. [7], in addition to the stacking interactions of Y56 with the phenolic ring of the substrate and the hydrogen bonding pattern of residue Y256, the hydroxyl groups in Y81 and S283 of MpDBR may also facilitate substrate binding, whereas L82 and F291 do not contain the corresponding hydroxyl groups in MpMDBRL (see Figure 5).

### 2.4. Transcript Abundance of DBRs and Their Response to Phytohormone Treatment

**Figure 6:**
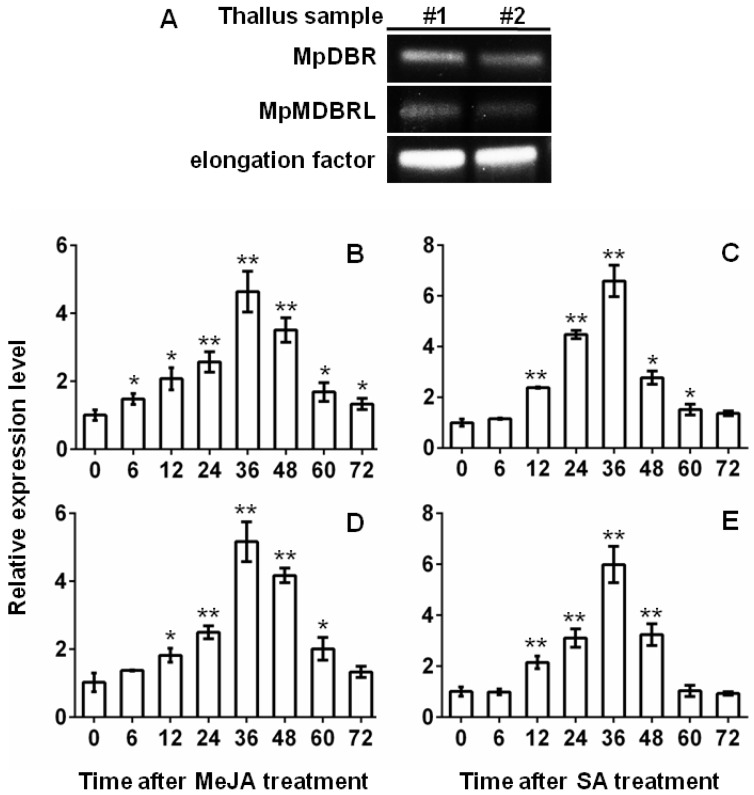
The transcript abundance of MpDBR and MpMDBRL in the thallus of *M. paleacea*. (**A**) MpDBR and MpMDBRL transcript abundance in two samples of *M. paleacea* thallus by sqRT-PCR. #1, #2: two selected thallus sample individuals. (**B**–**E**) Expression patterns of (**B**,**C**) MpDBR, (**D**,**E**) MpMDBRL in response to (**B**,**D**) MeJA, (**C**,**E**) SA at different time points (0, 6, 12, 24, 36, 48, 60, 72 h). Data are shown in the form mean ± SD (*n* = 3). *, **: means differ significantly from the level of sample at *t* = 0 h, respectively, *p* < 0.05 and <0.01.

The transcript abundance of the two *DBR* genes was determined in the thallus of *M. paleacea* by semi-quantitative RT-PCR (sqRT-PCR) analysis. The results showed that MpDBR and MpMDBRL were both clearly expressed in the thallus tissue (see Figure 6A). DBR is known for the plant protection against stress conditions. Transgenic tobacco plants had much higher 2-alkenal reductase activity levels and exhibited significantly less damage from treatment with methyl viologen plus light, or intense light [24].The plant hormonesmethyl jasmonate(MeJA) and salicylic acid (SA) play key roles in the response to stress [25,26]. Quantitative real-time PCR (qRT-PCR) analyses were carried out to determine the transcript abundance patterns of the *DBR* genes when the plant material was challenged by treatment with MeJA or SA. The abundance of MpDBR and MpMDBRL transcripts in the thallus increased slightly after 6 h of exposure to either MeJA or SA, peaking after 36 h, whereas the peak abundance of both *DBR* genes induced by MeJA was more than fourfold the background level, while that induced by SA treatment was over six fold the background level. The gene transcription level began to decline sharply at 60 h (see Figure 6B–E). In conclusion, similar to MpDBR, MpMDBRL also significantly responded to MeJA and SA treatment, providing evidence that the characterized MpMDBRL protein function as a genuine DBR in *M. paleacea* in plant defense, just as some *SmMTPSL* genes were induced by alamethicin treatment to emit terpenes [20].

The existence of two types of DBRs in *M. paleacea* poses a question regarding their evolutionary origins. The close similarity of MpDBR to the DBRs from other plants indicates that they are probably derived from a common ancestral plant DBR gene. However, MpMDBRL is likely to have a different evolutionary origin based on its close relationship to microbial DBRs. One hypothesis may be that an ancestral gene for MpMDBRL was acquired by *M. paleacea* or its recent ancestor from microbes through horizontal gene transfer [14], which was perhaps facilitated by the thalloid liverworts growth habit of being in intimate contact with the soil [13].

## 3. Materials and Methods

### 3.1. Chemicals and Reagents

The chemicals of *p*-coumaryl aldehyde, caffeyl aldehyde, and 5-hydroxyconiferyl aldehyde were obtained from our laboratory synthesized before [27]. *p*-Dihydrocoumaryl aldehyde, dihydrocaffeyl aldehyde, dihydroconiferyl aldehyde, 5-hydroxydihydroconiferyl aldehyde, and dihydrosinapyl aldehyde were all synthesized from their unsaturated form by reduction with hydrogen in the presence of Pd/C [22]. Coniferyl aldehyde and sinapyl aldehyde were purchased from Alfa Aesar (Heysham, UK). All the other reagents and solvents used were purchased from Sigma-Aldrich (St. Louis, MO, USA).

### 3.2. The Isolation and Analysis of DBR Sequences

The MpDBR full-length sequence and fragment of the MpMDBRL coding region sequence were obtained from a *M. paleacea* transcriptome sequencing database (SRP078650). *M. paleacea* thalli were maintained in a greenhouse held at 22 °C supplying a 12 h photoperiod. Total RNA was extracted using an RNAprep pure plant kit (Tiangen, Beijing, China). The full-length cDNA sequence of MpMDBRL was derived using the 3′-RACE and 5′-RACE technique, based on a Smarter™ Race cDNA amplification kit (Clontech, Mountain View, CA, USA) with the primers MpMDBRLGSP1 and MpMDBRLGSP2, respectively (see Table S1). Both full-length gene sequences were PCR-amplified using the primer pairs MpDBR-qF/R and MpMDBRL-qF/R (see Table S1). The resulting amplicons were purified, inserted into pMD19-T (Takara, Shiga, Japan) and sequenced.

### 3.3. Sequence Alignment and Phylogenetic Analysis

The deduced MpDBR and MpMDBRL polypeptides were aligned with known DBRs from other plants using DNAMAN v5.2.2 software (LynnonBiosoft, Vaudreuil, QC, Canada). A neighbor-joining (NJ) phylogenetic tree was constructed with a collection of DBRs using the MEGA v4.0 software [28] and the strength of the relationships was quantified using a 1000 replicate bootstrap analysis. The Swiss-model (http://swissmodel.expasy.org) was adopted to achieve the homology modelings of MpDBR and MpMDBRL, based on the 2J3J structure of *A. thaliana* DBR bound to *p*-coumaryl aldehyde and NADP^+^ [7]. The models were visualized by PyMOL software (www.pymol.org/citing).

### 3.4. Recombinant Protein Expression and Purification

The open reading frames (ORFs) of MpDBR and MpMDBRL were amplified from the two cDNA clones using, respectively, primer pairs MpDBR-F/R and MpMDBRL-F/R (see Table S1). The resulting two amplicons were each inserted into a pET32a plasmid (Novagen, Darmstadt, Germany) and then introduced into the *E. coli* strain BL21 (DE3). Expression was induced by the addition of 0.5 mM isopropyl β-d-1-thiogalactopyranoside and incubation at 16 °C for 12 h, and the proteins were purified as described previously [8]. Protein quality was achieved by analysis of SDS-PAGE separations and quantification was achieved using the Bradford assay (Bio RadLaboratories, Hercules, CA, USA) with bovine serum albumin used as the standard.

### 3.5. Enzyme Assays

Each 50 μL assay comprised of 5 μg purified protein, 200 μM substrate, 1 mM NADPH, formulated in 100 mM potassium phosphate buffer, pH 6.5. The reactions were initiated by the addition of the protein and terminated after 30 min by the addition of a double volume of ethyl acetate. The ethyl acetate fraction evaporated and the residue dissolved in 50 μL methanol. The methanol solution was separated by reverse phase HPLC using a 5 μm XDB-18 column (Agilent Technologies, Palo Alto, CA, USA), applying a flow of 0.8 mL min^−1^. The mobile phase was varied linearly over 20 min from 5:95 to 30:70 of acetonitrile:aqueous 1% glacial acetic acid. The enzymatic products were identified by each authentic standard and by mass spectrometry using aliquid chromatograph mass spectrometer-2020 device (Shimadzu Corporation, Kyoto, Japan). The effect on enzymatic activity of altering the solution pH over the range 5.0–8.0 was monitored by running the reactions at 30 °C for 30 min in a range of buffers. The optimal temperature was determined in reactions formulated to a pH of 6.5. The quantity of reaction product present was estimated from a standard calibration curve.

### 3.6. Plant Phytohormone Treatment and Gene Expression Profiling

Two-month-old thalli were exposed to either 100 μM MeJA or 100 μM SA for 0, 6, 12, 24, 36, 48, 60, and 72 h to assess their phytohormonal response. Total RNA was extracted and the first cDNA strand was generated from a 1 μg aliquot of RNA using a PrimeScript RT Master Mix kit (Takara), and this was subsequently used as the template for PCRs used to assess transcript abundance. A gene encoding a *M. paleacea* elongation factor was used as the reference sequence [29]. The segments of MpDBR and MpMDBRL sequence were amplified using, respectively, the primer pair MpDBR-RTF/R and MpMDBRL-RTF/R (see Table S1) via a sqRT-PCR or a qRT-PCR assay. The qRT-PCRs were based on PrimeSTAR^®^Max DNA Polymerase (Takara) using the manufacturer’s protocol.

### 3.7. Sequence Accession Numbers

The sequence data required for this research were recovered from GenBank. PtPPDBR (DQ829775), AaDBR1 (FJ750460), RiRZS1 (JN166691), NtDBR (AB036735), PulR (AY300163), AtDBR1(BT022058), HvALH (AY904340), PaDBR1 (KF051271), PaDBR2 (KF051272), RiALR (PKC04648), RiAOR (GBC14596), DrLTB4DH (PBP21645), BmAOR (ORX66992), LcLTB4DH (CDH57485).

## 4. Conclusions

Here, two liverwort DBRs were characterized. The data from alignment analysis, phylogeneticanalysis, enzyme assays, and expression patterns analysis indicate that the *M. paleacea* genome contains two distinct types of active DBRs, with MpMDBRL reported as the first characterized microbial type of DBR to occur in a plant. This study facilitates the reconstruction of the evolution of this important plant protein family.

## Figures and Tables

**Table 1:** The substrate specific activity of MpDBR and MpMDBRL from *M. paleacea*.

Substrate	Structure	Specific Activity (nmol mg^−^^1^ min^−^^1^)	
		MpDBR	MpMDBRL
*p*-Coumaryl aldehyde	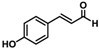	29.08 ± 1.48	21.26 ± 1.46
Caffeyl aldehyde	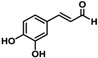	29.60 ± 1.35	25.88 ± 0.88
Coniferyl aldehyde	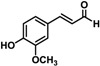	28.61 ± 0.69	20.17 ± 0.48
5-Hydroxyconiferyl aldehyde	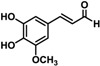	26.87 ± 2.21	21.34 ± 0.54
Sinapyl aldehyde	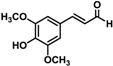	ND ^a^	ND

^a^ no detectable activity.

## Supplementary Materials

The following are available online: Figure S1, Table S1.
