# Supplementary material for: Molecular Diversity of Alkenal Double Bond Reductases in the Liverwort Marchantia paleacea
Source: Molecules. 2018 Jul 4;23(7):1630. doi: 10.3390/molecules23071630 (PMC6099575; doi:10.3390/molecules23071630)
Supplement: Supplementary file 1 [file molecules-23-01630-s001.pdf]

Molecular Diversity of Alkenal Double Bond Reductases in the Liverwort *Marchantia paleacea*

Yi-Feng Wu, Hong-Bo Zheng, Xin-Yan Liu, Ai-Xia Cheng*, Hong-Xiang Lou*

Key Laboratory of Chemical Biology of Natural Products, Ministry of Education, School of Pharmaceutical Sciences, Shandong University, Jinan, 250012, China

* Correspondence: louhongxiang@sdu.edu.cn （H.L.）; Fax, +86 531 88382019 (H.L.); Aixia Cheng, E-mail, aixiacheng@sdu.edu.cn (A.C.); Fax, +86 531 88382019 (A.C.)

Supplementary Materials:

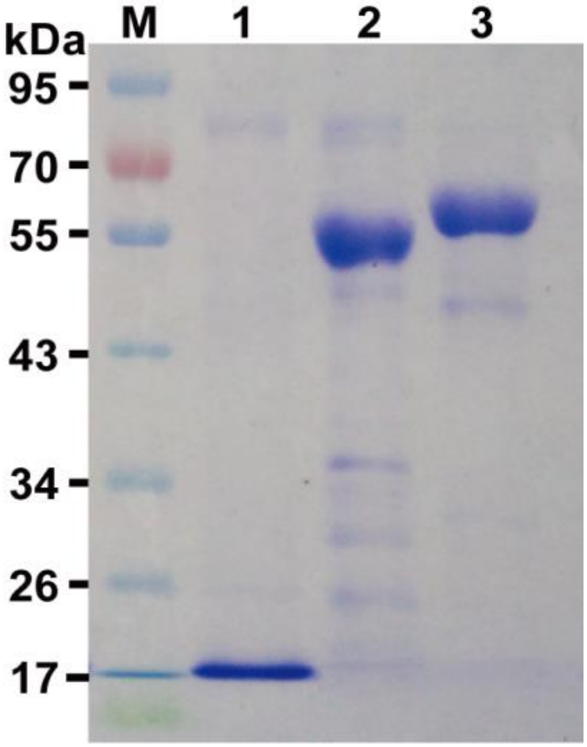

Figure S1. SDS-PAGE analysis of recombinant DBR proteins. M: molecular mass standards. Lanes 1-3: Purified proteins expressed in *E. coli* BL21 cells harboring (1) an empty pET32a plasmid, (2) pET32a-MpDBR, (3) pET32a-MpMDBRL.

Table S1. Sequences of PCR primers used.

Primer name	Primer sequences (5' to 3')
MpDBR-qF	CGAAATCCTCGTCACATCCG
MpDBR-qR	GGTCTCACCTTCCGCTAAAC
MpMDBRLGSP1	GAGCCCCACATTAAATCATCTTTACCAC
MpMDBRLGSP2	TTTACAACTTCTGAAACTCCCAAACCAT
MpMDBRL-qF	ACGCGGGATCGAACAAAG
MpMDBRL-qR	TCAACAACTAGTAATCAGCAATC
MpDBR-F	GGGGTACCATGGCCGGAACCGAGGTAAC
MpDBR-R	CGGGATCCCTAAAAATCTGCAACTTTTA
MpMDBRL-F	CGGGATCCATGTACGCGGGATCGAACAAA
MpMDBRL-R	CCCTCGAGGTAATCAGCAATCTTTACAA
MpDBR-RTF	AAAAGGCATTGTCCACAGGG
MpDBR-RTR	CCATCGGCTATATCCTCCACG
MpMDBRL-RTF	GATTAACCTGCTATGCGTCCC
MpMDBRL-RTR	GGACAAAGTTCGGATAATGCC
Elongation factor F	CATCGCGCTGTGGAAATTCG
Elongation factor R	TCATCTGCTTCACTCCCAGC
